# Handheld Real-Time LED-Based Photoacoustic and Ultrasound Imaging System for Accurate Visualization of Clinical Metal Needles and Superficial Vasculature to Guide Minimally Invasive Procedures

**DOI:** 10.3390/s18051394

**Published:** 2018-05-01

**Authors:** Wenfeng Xia, Mithun Kuniyil Ajith Singh, Efthymios Maneas, Naoto Sato, Yusuke Shigeta, Toshitaka Agano, Sebastian Ourselin, Simeon J. West, Adrien E. Desjardins

**Affiliations:** 1 Wellcome/EPSRC Centre for Interventional and Surgical Sciences, University College London, Charles Bell House, 67-73 Riding House Street, London W1W 7EJ, UK; efthymios.maneas.14@ucl.ac.uk (E.M.); s.ourselin@ucl.ac.uk (S.O.); a.desjardins@ucl.ac.uk (A.E.D.); 2 Department of Medical Physics and Biomedical Engineering, University College London, Gower Street, London WC1E 6BT, UK; 3 Research and Business Development Division, PreXion Corporation, Stationsplein 45 A4.004, 3013AK Rotterdam, The Netherlands; 4 Research and Development Division, 1-14-1, Kandasudacho, Chiyoda-ku, Tokyo 101-0041, Japan; saton@prexion.co.jp (N.S.); shigeta@prexion.co.jp (Y.S.); agano@prexion.co.jp (T.A.); 5 Centre for Medical Imaging Computing, University College London, Gower Street, London WC1E 6BT, UK; 6 Department of Anaesthesia, University College Hospital, Main Theatres, Maple Bridge Link Corridor, Podium 3, 235 Euston Road, London NW1 2BU, UK; simeon.west@nhs.net

**Keywords:** photoacoustic imaging, ultrasonography, LED, needle guidance, vasculature, minimally invasive procedures

## Abstract

Ultrasound imaging is widely used to guide minimally invasive procedures, but the visualization of the invasive medical device and the procedure’s target is often challenging. Photoacoustic imaging has shown great promise for guiding minimally invasive procedures, but clinical translation of this technology has often been limited by bulky and expensive excitation sources. In this work, we demonstrate the feasibility of guiding minimally invasive procedures using a dual-mode photoacoustic and ultrasound imaging system with excitation from compact arrays of light-emitting diodes (LEDs) at 850 nm. Three validation experiments were performed. First, clinical metal needles inserted into biological tissue were imaged. Second, the imaging depth of the system was characterized using a blood-vessel-mimicking phantom. Third, the superficial vasculature in human volunteers was imaged. It was found that photoacoustic imaging enabled needle visualization with signal-to-noise ratios that were 1.2 to 2.2 times higher than those obtained with ultrasound imaging, over insertion angles of 26 to 51 degrees. With the blood vessel mimicking phantom, the maximum imaging depth was 38 mm. The superficial vasculature of a human middle finger and a human wrist were clearly visualized in real-time. We conclude that the LED-based system is promising for guiding minimally invasive procedures with peripheral tissue targets.

## 1. Introduction

Precise and efficient device guidance is critically important for minimally invasive procedures in many clinical fields such as fetal medicine, regional anesthesia and pain management, and interventional oncology [1,2]. Successful procedural outcomes are dependent on the accurate visualization of both the invasive medical device and the procedure target. Ultrasound (US) imaging is commonly used for image guidance, as it provides high-resolution anatomical images in real-time. However, US visualization of invasive medical devices such as metal needles can be challenging [3]. For example, when the needle is inserted into tissue at a steep angle, the US waves may be reflected away from the aperture of the US imaging probe, such that the needle effectively becomes invisible. Additionally, US imaging provides insufficient contrast to robustly differentiate tissue targets from their surroundings in many minimally invasive procedures. A loss of visibility of the needle or the tissue target can result in serious complications [4].

Photoacoustic (PA) imaging is a hybrid imaging modality that offers optical spectroscopic contrast and ultrasonic resolution. In PA imaging, pulsed excitation light is absorbed in tissue, and the resulting US waves are received and processed to reconstruct an image [5,6]. PA imaging has emerged as a powerful biomedical imaging modality in the last decade with tremendous potential for a wide range of clinical and preclinical applications [7,8,9,10,11,12,13,14,15,16,17]. PA imaging can be readily combined with US imaging using a clinical US imaging probe; with the same transducer elements in the probe used for reception, the images are naturally co-registered. These dual-mode photoacoustic and ultrasound (PA/US) imaging systems provide both structural and molecular contrast [17,18,19,20].

The use of dual-mode PA/US imaging with a clinical US imaging probe for guiding minimally invasive procedures has attracted increasing interest [21,22,23,24,25,26,27,28,29,30,31]. Kim et al. reported a PA/US imaging system for guiding sentinel lymph node biopsies, with light delivered to the tissue surface via a bifurcated optical fiber bundle integrated with the US imaging probe [21]. Similarly, with surface-based light illumination, Su et al. demonstrated the visualization of clinical metal needles with PA imaging [22]. Surface-based illumination provides wide-field tissue imaging, but it suffers from a rapid reduction of PA signal amplitude with an increase in tissue depth. Interventional PA imaging addresses this limitation with a complementary light delivery approach: PA excitation light is delivered inside tissue through an optical fiber integrated within an invasive medical device. Interventional PA imaging has a strong potential to guide minimally invasive procedures in different clinical contexts such as fetal surgery [23,24], regional anesthesia and interventional pain management [25,26], breast biopsies [27], prostate brachytherapy [28,29,30], and neurosurgery [31].

Compact laser diodes and light-emitting diodes (LEDs) can be used as PA excitation sources [32,33,34,35,36,37,38,39,40], and their small size and low cost relative to some conventional sources may facilitate the clinical translation of the technology [41]. Whilst their pulse energies are typically much lower than those of conventional excitation sources, their pulse repetition frequencies (PRFs) can be higher, so that frame-to-frame averaging and coded excitation sequences can be used to increase the signal-to-noise ratio (SNR).

In this work, the use of an LED-based PA/US imaging system for guiding minimally invasive procedures was investigated for the first time. The performance of this system for visualizing clinical metal needles inserted at various angles and tissue targets at different depths was evaluated with ex vivo tissue phantoms. Initial indications of the potential of the system for guiding peripheral procedures were obtained by imaging the superficial vasculature in the fingers and the wrists of human volunteers.

## 2. Materials and Methods

### 2.1. System Description

**Figure 1:**
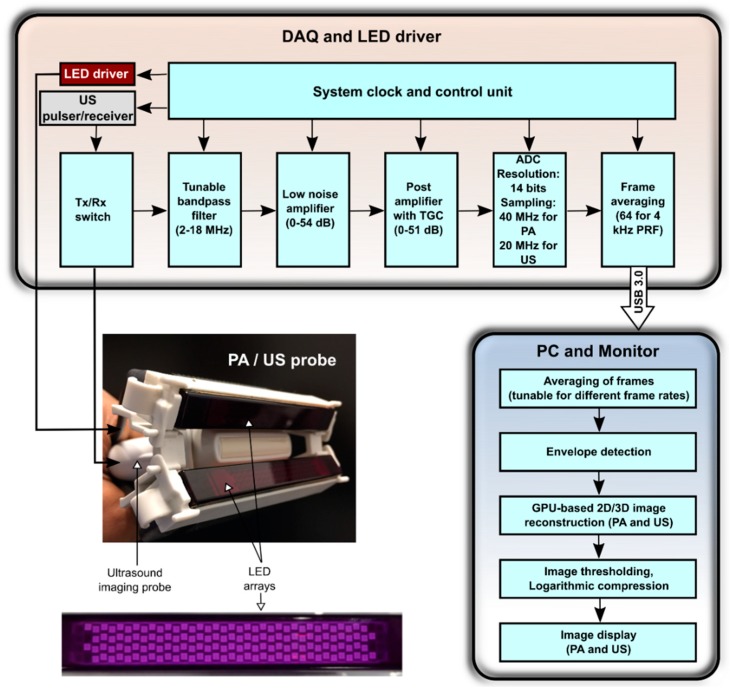
Block diagram of the light-emitting diode (LED)-based photoacoustic (PA) and ultrasound (US) imaging system. Middle left: photograph of the arrangement of the two LED arrays and the US imaging probe. The two arrays are positioned on both sides of the US imaging probe, angled towards the imaging plane. Bottom left: photograph of an LED array (wavelength: 850 nm). It consists of four rows of 36 LEDs (dimensions: 1 mm × 1 mm). DAQ: data acquisition; Tx: transmit; Rx: receive; TGC: time gain compensation; ADC: analog-to-digital converter; PRF: pulse repetition frequency; USB: universal serial bus; PC: personal computer; GPU: graphics processing unit.

AcousticX (PreXion Corporation, Tokyo, Japan) is a commercially available LED-based PA/US system which can acquire interleaved PA and US images and display them in real-time. Figure 1 shows the block diagram of AcousticX in which data and processing flow is detailed.

#### 2.1.1. Light Illumination

The PA/US imaging system uses LED arrays to deliver PA excitation light with a wavelength of 850 nm. Each array comprises four rows of LEDs, with 36 LEDs in each row; the size of each LED is 1 mm × 1 mm. Each array can deliver a maximum optical energy of 200 µJ per pulse. The peak current flow for each LED array is 20 A, and the LEDs can be driven at a PRF of 1 KHz to 4 KHz. The LED pulse duration is tunable over the range of 30 ns to 100 ns. In this study, a pulse duration of 70 ns was used to maximize the PA efficiency (signal strength/light pulse energy). The optical energy was dependent only on the driving voltage and the pulse width, and there was no relation between the PRF and the optical output power of LEDs within the range of PRFs considered in this study (data not shown). One LED array was affixed on each side of a linear-array US imaging probe so that the light beams from the two LED arrays overlapped at the focus depth of this probe. In the absence of optical scattering, the overlap region was approximately 50 mm × 7 mm in the YX-plane, with a maximum fluence of 0.11 mJ/cm^2^.

#### 2.1.2. Image Acquisition and Processing

US reception was performed with a linear-array lead zirconate titanate (PZT)-based US probe that comprises 128 elements over a distance of 38.4 mm. Each element has a transverse length of 5 mm, a pitch of 0.3 mm, a central frequency of 9 MHz, and a measured −6 dB bandwidth of 77%. The array incorporates an acoustic lens to achieve an elevational focus of 15 mm.

For both PA and US imaging, RF data from all US transducer elements were acquired from 128 channels at sampling rates of 40 MHz and 20 MHz respectively, and the data were then transferred to the graphics processing unit (GPU) board using a USB 3.0 interface. US imaging was performed with single-angle plane-wave US transmissions, so that one B-mode US image was obtained for each plane-wave transmission. Likewise, one PA image was obtained for each pulse of excitation light. The acquired US and PA data were averaged across sequentially acquired PA images, reconstructed using an inbuilt GPU-based Fourier-domain reconstruction algorithm, and then displayed in real-time on a high-resolution monitor (Figure 1). Image thresholding was varied manually in real-time. The frame rate was dependent on the number of averages: when averaging was performed across 2560 images, the frame rate was 1.5 frames per second (FPS); when averaging was performed across 128 images, the frame rate was 30 FPS. A maximum of 1536 PA frames and 1536 US frames (imaging depth: 4 cm) can be stored in memory at one time. The raw RF data were saved and made available for offline Fourier-domain reconstruction [42].

### 2.2. Spatial Resolution Measurements

**Figure 2:**
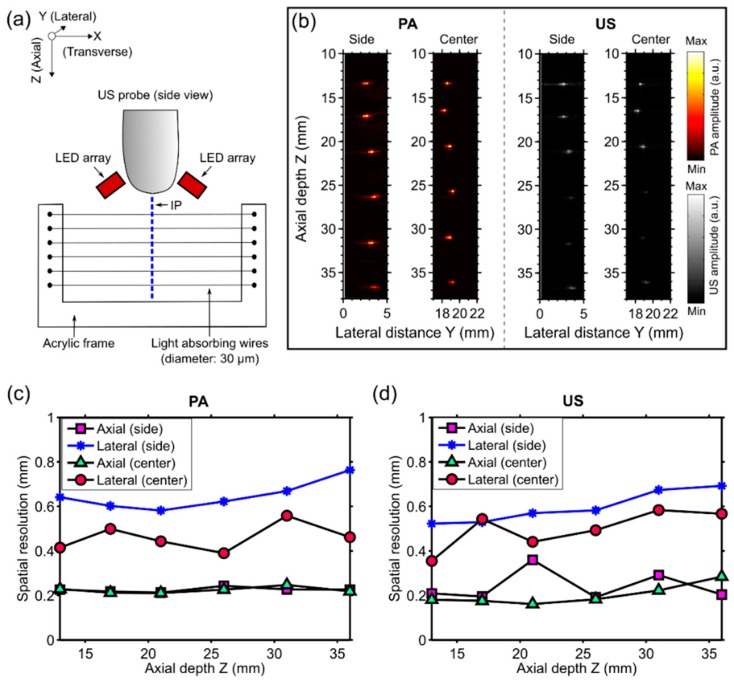
(**a**) Schematic illustration of the resolution phantom and the measurement geometry. The phantom, which comprised six light-absorbing wires mounted at different depths (Z) on an acrylic frame, was positioned so that these wires were perpendicular to the imaging plane (IP). LED: light-emitting diode; (**b**) Photoacoustic (PA) and ultrasound (US) images were acquired with the phantom at two different lateral (Y) positions so that the wires were near the side and the center with respect to the ultrasound imaging probe, respectively. Both PA and US images were displayed in linear scales (a.u.: arbitrary units); (**c**) Measured axial and lateral PA imaging resolution for the two lateral positions of the phantom (side and center) as a function of depth; and (**d**) the corresponding values for US imaging.

The spatial resolution of the dual-mode PA/US imaging system was measured by imaging a resolution phantom that comprised six light-absorbing wires (diameter: ca. 30 µm) as targets. The wires were fabricated by coating tungsten wires (diameter: ca. 25 µm) with a carbon black paint marker (no. 4610 337, Liquitex, Cincinnati, OH, USA) that were mounted on a U-shaped acrylic frame so that they were parallel to each other (Figure 2a). The distances between each pair of neighboring wires were approximately 5 mm.

Imaging was performed with both the resolution phantom and the imaging probe in water. The phantom (acrylic frame with the light-absorbing wires) was arranged so that the wires were perpendicular to the imaging plane and intersected at approximately the same lateral (Y) positon and at six different depths (Z) (Figure 2a,b). Raw PA and US data with the wires at two different lateral positions (side and center with respect to the ultrasound imaging probe) were acquired, and images were reconstructed offline. To calculate the spatial resolution for both PA and US images, axial and lateral profiles across the local maxima that corresponded to the wires were plotted, and the full width at half maximum (FWHM) values yielded the axial and lateral resolutions, respectively.

### 2.3. PA and US Imaging of Clinical Metal Needle Insertions

To demonstrate the potential of the system for visualizing clinical metal needles, a 14 gauge needle (Terumo, Surrey, UK) was inserted into chicken breast tissue ex vivo at different angles. At each angle, the signal-to-noise ratio (SNR) of the needle shaft was calculated for both the US and the PA images.

The SNR was defined as SNR = S/σ, where S is the mean of the image amplitude in a signal region, and σ is the standard deviation of the image amplitude in a noise region. The signal region was empirically chosen as a rectangular region where the PA signal was apparent; the noise region was manually chosen to correspond to a rectangular region away from the needle. The same signal and noise regions were used to calculate the SNR for both the PA and the US images.

### 2.4. PA Imaging of a Blood-Vessel-Mimicking Phantom

To evaluate the sensitivity with which PA imaging can be performed, a blood-vessel-mimicking phantom that was created by embedding cylindrical vessels (5 mm diameter) into chicken breast at different depths was used. The vessels comprised gel wax (Mindsets Online, Waltham Cross, UK), with carbon black ink (carbon black, Cranfield Colours, Cwmbran, UK) and TiO_2_ particles (13463-67-7, ReagentPlus 99%, Sigma-Aldrich, St. Louis, MO, USA) for optical absorption and scattering, respectively [43,44]. The optical absorption coefficient was 1 mm^−1^ at 850 nm; the optical reduced scattering coefficient was 0.5 mm^−1^. The PA/US probe was aligned in such a way that the vessels were perpendicular to the imaging plane. Several measurements were performed by adding multiple layers of chicken tissue and thereby increasing the depth of the vessel relative to the probe surface. The PA and US data of all measurements were saved for further processing. The SNR was calculated for the PA and the US images that were reconstructed offline for seven different depths that ranged from 12 mm to 38 mm and for four frame rates that ranged from 1.5 to 30 FPS. These calculations were performed in the same way as those in Section 2.3, where the signal region was empirically chosen as a rectangular region that enclosed the vessel.

To obtain a preliminary indication of the potential of our system to guide percutaneous procedures, PA/US imaging was performed while a 14 gauge needle was inserted into a blood-vessel-mimicking phantom. This phantom was the same type as that used for the sensitivity evaluation, with the gel wax vessel positioned at a depth of 2 cm. PA and US image pairs were displayed at 10 Hz, and reconstructed images and videos were saved in real-time.

### 2.5. In Vivo Imaging

To demonstrate the potential of our system to image superficial vasculature in vivo, a middle finger of a healthy volunteer was imaged with the imaging probe held manually and the finger positioned in water, interleaved PA and US imaging from the side of the finger comprising one of the digital arteries was performed, with images from each modality acquired at ten FPS. A real-time image display was useful to align the imaging probe in so that the pulsating radial artery was visible.

The same volunteer’s right wrist was scanned in cross-section. The protocol was similar to the protocol used to image the middle finger, except that ultrasound gel was used instead of water as a coupling medium, and a lower frame rate was used (six FPS).

## 3. Results

### 3.1. Spatial Resolution

The spatial resolutions of PA and US imaging were similar (Figure 2c,d). The axial resolution was consistent at the center and the side (Y direction), and it was over the measured depth (Z) range of 13 to 36 mm. For PA imaging, the mean axial resolution was 0.22 mm with a standard deviation of 0.01 mm, with statistics obtained from all measured Y and Z positions; for US imaging, the mean axial resolution was 0.22 ± 0.06 mm. The lateral resolution of both PA and US images was better at the center of the imaging plane than at the side. At the center, the lateral resolution of PA images was relatively consistent (0.46 ± 0.06 mm); with US imaging, it increased with Z from 0.35 mm to 0.57 mm. At the side, the lateral resolution of both PA and US images increased with Z, from 0.64 mm to 0.76 mm and from 0.52 mm to 0.69 mm, respectively.

### 3.2. Angle Dependence of US and PA Imaging for In-Plane Needle Insertions

**Figure 3:**
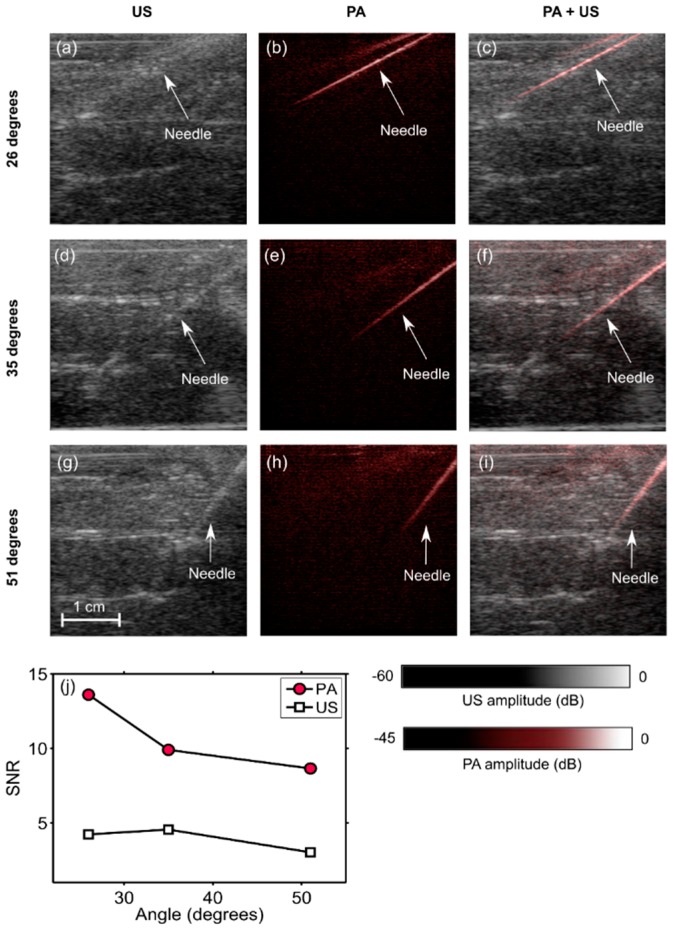
Photoacoustic (PA) images, ultrasound (US) images, and overlaid PA and US images (PA + US) of a spinal needle inserted into chicken breast tissue at different angles (**a**–**i**). Signal-to-noise (SNR) ratios for PA images were substantially higher than those for US images at all insertion angles (**j**). During the insertions, these images were reconstructed and displayed in real-time on a logarithmic scale. Here, they are presented without the uppermost 5 mm, which contained the ultrasound gel. Each point in the SNR plots was calculated from one spatial region.

When inserted into the chicken breast tissue, the needle was barely visible with US imaging (Figure 3a,d,g), and clearly visible with PA imaging (Figure 3b,e,h). The needle was visible with PA imaging at depths of up to 2 cm. The spatial locations of the needle observed on US images corresponded well to those observed on PA images, as evidenced by the overlays (Figure 3c,f,i). The SNR values for PA images were substantially higher than those for US images. With both US and PA imaging, the SNR decreased as the needle insertion angle increased (Figure 3j).

### 3.3. Imaging Depth—Phantom Experiment

**Figure 4:**
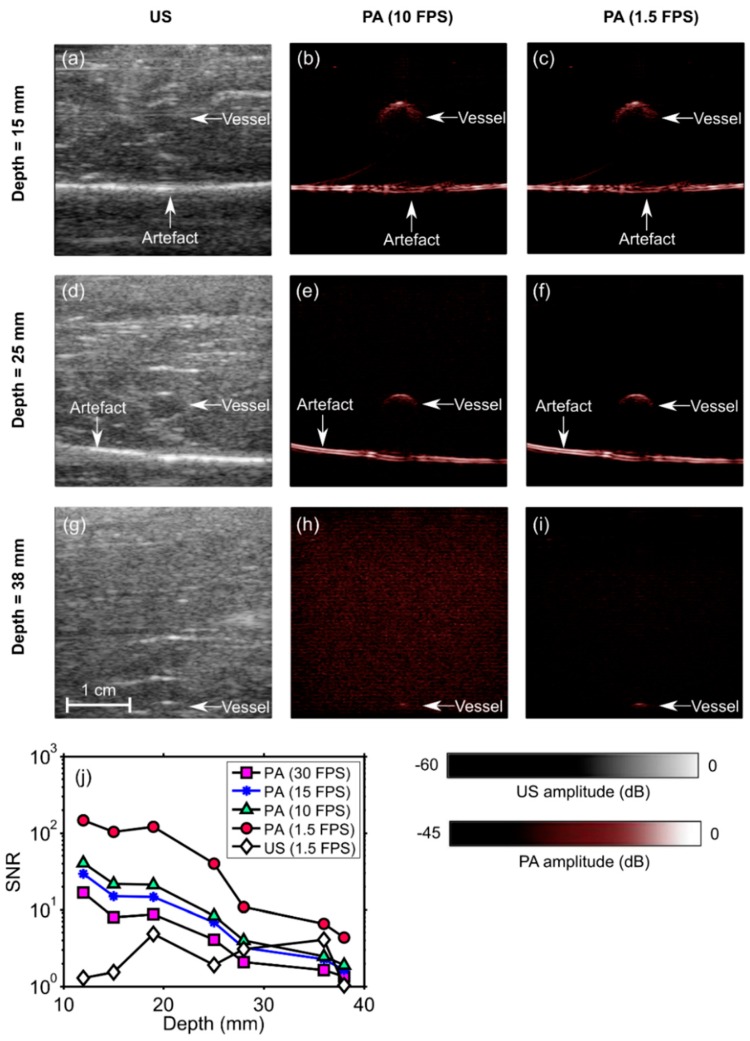
Photoacoustic (PA) and ultrasound (US) images of a phantom comprising a vessel positioned in chicken breast tissue at different depths (**a**–**i**). The signal-to-noise ratio (SNR) of the PA images decreased with the vessel depth and with the imaging frame rate (**j**). At 1.5 frames per second (FPS), the SNR of the US images was lower than that of the PA images for all depths. During the insertions, these images were reconstructed and displayed in real-time on a logarithmic scale. Here, they are presented without the uppermost 5 mm, which contained the ultrasound gel. FPS: frames per second. Each point in the SNR plots was calculated from one spatial region.

**Figure 5:**
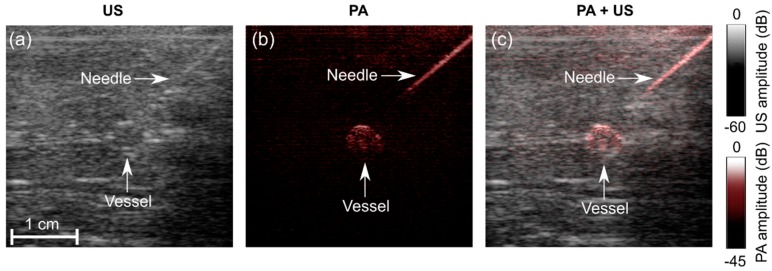
Photoacoustic (PA) and ultrasound (US) imaging of a needle inserted towards a vessel mimicking phantom. (**a**) US image; (**b**) PA image at 850 nm; (**c**) PA + US image overlay. During the insertions, these images were reconstructed and displayed in real-time on a logarithmic scale. Here, they are presented without the uppermost 5 mm, which contained the ultrasound gel. A video is provided in Supplementary Materials (Video S1).

With the blood-vessel-mimicking phantom, the vessel had a much more prominent appearance with PA imaging than with US imaging. The SNR of the PA images decreased with the depth at which the vessel was positioned in the chicken breast and also with the frame rate (Figure 4). This vessel was apparent with PA imaging at depths of up to 2.8 cm, with a frame rate of 30 Hz. When the frame rate was decreased to 10 Hz, the vessel was apparent at depths of up to 3.8 cm. At 1.5 FPS, the SNR of the US images was lower than that of the PA images at all depths. An image artefact of unknown origin (horizontal line indicated in the images) was also apparent in both US and PA images. With the needle inserted towards the vessel, the visibility of both the needle and the vessel was low with US imaging (Figure 5a) and high with PA imaging (Figure 5b). A video obtained from real-time reconstruction as the needle was inserted into the vessel is provided in the Supplementary Materials (Video S1).

### 3.4. In Vivo Imaging

**Figure 6:**
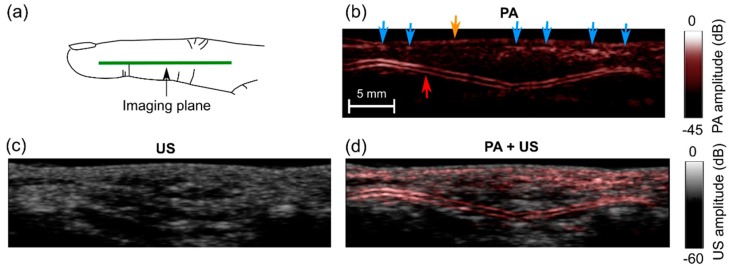
Photoacoustic (PA) and ultrasound (US) images of a middle finger of a human volunteer. (**a**) Schematic indicating the location of the imaging plane; (**b**) PA image; (**c**) US image; (**d**) PA + US image overlay. In the PA image (**b**), signals from low depths may have corresponded to veins (blue arrows) and the skin surface (yellow arrow); signals from a two-layered structure may have corresponded to a digital artery (red arrow). These images were reconstructed and displayed in real-time on logarithmic scales. Here, they are presented without the uppermost 5 mm, which contained the water. Pulsations of the digital artery were apparent (Supplementary Materials; Video S2).

While imaging the human finger, high PA signals were observed from a two-layered subsurface structure (Figure 6b). With a real-time image display, pulsations were readily apparent (Supplementary Materials; Video S2), which suggests that the subsurface structure was a digital artery. Additional subsurface signals were observed, which likely corresponded to smaller vessels such as superficial veins (blue arrows). During imaging, the finger was slightly compressed, and this may be the reason why the veins were not visible as two-layered features. Visually, there was a fairly strong correspondence between the locations of the PA signals and the appearances of the US images. However, PA and US images appeared to provide distinct information; PA signals were not present at all locations where there were features in the US images and vice-versa.

**Figure 7:**
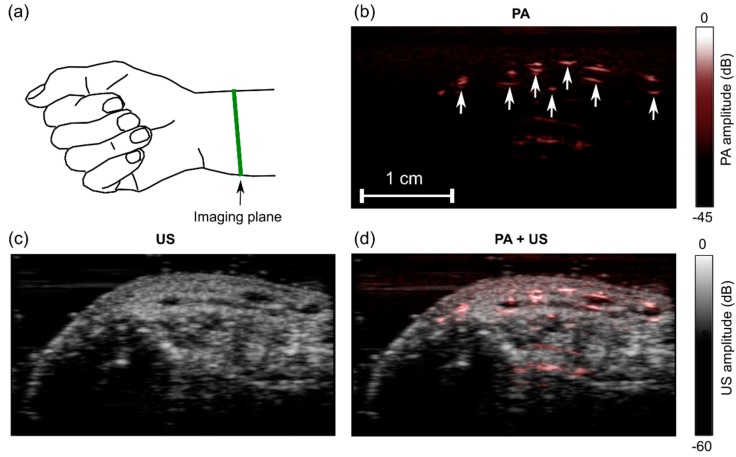
Photoacoustic (PA) and ultrasound (US) images of a wrist of a human volunteer. (**a**) Schematic indicating the location of the imaging plane; (**b**) PA image; (**c**) US image; (**d**) PA + US image overlay. In the PA image (**b**), the prominent signals likely originated from blood vessels (arrows). Images were reconstructed and displayed in real-time on logarithmic scales. Here, they are presented without the uppermost 5 mm, which contained the ultrasound gel. Bulk tissue motion and localized pulsatile subsurface motion were apparent in the corresponding video (Supplementary Materials; Video S3).

With the human wrist, the PA image comprised multiple regions with high signal values (Figure 7b). These regions likely originated from superficial blood vessels. They corresponded well to anechoic regions on the US image (Figure 7c,d). Bulk tissue motion and localized subsurface motion were clearly visible in real-time with both PA and US imaging (Supplementary Materials; Video S3). Despite this motion and the use of signal averaging for PA imaging, there were no visible fluctuations in the PA signal amplitudes.

## 4. Discussion and Conclusions

For the clinical translation of PA imaging to guide peripheral minimally invasive procedures, it will likely be important to have compact excitation light sources and real-time imaging capabilities for compatibility with current workflow. In this study, for the first time, we demonstrated the feasibility of visualizing clinical needles and a vascular target with real-time PA and US imaging using an LED-based PA system. This paradigm could be useful for guiding peripheral intravenous line placement, particularly in difficult cases that arise from diseases such as diabetes, intravenous drug abuse, and sickle cell disease [45].

There are several distinct advantages of using LEDs as PA excitation sources. First, as LEDs and their drivers are widely used in consumer electronics, they can benefit from cost reductions that arise from high-volume manufacturing. Second, as the frequency content of PA signals is dependent on the pulse width of light used for excitation, the pulse width of the LED light could be tuned to optimize imaging for different tissue depths and for different clinical contexts. Finally, coded excitation could be used to increase the SNR of generated PA signals [35,40]. In future implementations of this PA system, LED arrays could be fabricated on highly curved surfaces for endoscopic or vascular applications.

There are several ways in which the system could be improved. LED arrays could be fabricated with multiple wavelengths to perform multispectral PA imaging and thereby to provide spectroscopic tissue specificity. This functionality could be useful to differentiate arteries and veins based on differences in hemoglobin absorption spectra [23,24] and also to identify nerves based on differences in water and lipid absorption spectra [23,25,26]. Depending on their origin, horizontal PA artefacts could be mitigated by using the PAFUSion technique reported by Kuniyil Ajith Singh et al. [46,47,48] or with singular value decomposition [49]. US image quality could be improved using a sequence of electronically focused transmissions in place of plane-wave transmissions. In this study, the choice of plane-wave US transmissions was made to retain the high frame rate (PA + US) using parallel acquisition and processing. Further improvements in US image quality could be obtained with angular compounding and postprocessing speckle-reduction algorithms. With the LED side arrays positioned at an angle to the imaging plane and extending in depth beyond the surface of the ultrasound probe, it was often challenging to achieve acoustic coupling with ultrasound gel during in vivo human imaging. Future versions could include compact co-axial excitation schemes [50].

It is encouraging that the system used in this study could visualize both needles and vessel targets at cm-scale depths, considering the low pulse energies provided by the LED arrays. These pulse energies (200 µJ for each array) are approximately two orders of magnitude lower than those used in similar studies with Q-switched Nd:YAG lasers (typically >10 mJ) [41,49]. To further increase the depth at which medical devices and tissue targets could be visualized, excitation light could be coupled into an optical fiber to deliver light from within the tissue, in addition to the surface-based illumination provided by the LED arrays [23,24,25,26]. A system in which excitation light is delivered both at the surface and through an interventional device could provide both a large field of view and a large imaging depth.

The shape and the orientation of the needle were clearly visualized with PA imaging; however, an unambiguous determination of the needle tip position with this approach may be challenging. In the future, ultrasonic tracking could be combined with the current system, with a fiber-optic ultrasound receiver or transmitter within the needle being used to communicate with an external ultrasound array [51,52,53,54,55,56] and to deliver excitation light into tissue.

With the capability of providing real-time visualization of clinical metal needles and tissue targets, the LED-based PA/US system used in this study could be useful for guiding minimally invasive procedures in many clinical contexts.

## Supplementary Materials

The following are available online at http://www.mdpi.com/1424-8220/18/5/1394/s1, Video S1: Real-time photoacoustic and ultrasound visualization of a needle as it is inserted into a vessel phantom, Video S2: Real-time photoacoustic and ultrasound imaging of the finger of a human volunteer, Video S3: Real-time photoacoustic and ultrasound visualization of the wrist of a human volunteer.
